# Telomere DNA Deficiency Is Associated with Development of Human Embryonic Aneuploidy

**DOI:** 10.1371/journal.pgen.1002161

**Published:** 2011-06-30

**Authors:** Nathan R. Treff, Jing Su, Deanne Taylor, Richard T. Scott

**Affiliations:** 1 Reproductive Medicine Associates of New Jersey, Morristown, New Jersey, United States of America; 2 Robert Wood Johnson Medical School, University of Medicine and Dentistry of New Jersey, New Brunswick, New Jersey, United States of America; Washington State University, United States of America

## Abstract

Aneuploidy represents the most prevalent form of genetic instability found in human embryos and is the leading genetic cause of miscarriage and developmental delay in newborns. Telomere DNA deficiency is associated with genomic instability in somatic cells and may play a role in development of aneuploidy commonly found in female germ cells and human embryos. To test this hypothesis, we developed a method capable of quantifying telomere DNA in parallel with 24-chromosome aneuploidy screening from the same oocyte or embryo biopsy. Aneuploid human polar bodies possessed significantly less telomere DNA than euploid polar bodies from sibling oocytes (−3.07 fold, P = 0.016). This indicates that oocytes with telomere DNA deficiency are prone to aneuploidy development during meiosis. Aneuploid embryonic cells also possessed significantly less telomere DNA than euploid embryonic cells at the cleavage stage (−2.60 fold, *P* = 0.002) but not at the blastocyst stage (−1.18 fold, *P* = 0.340). The lack of a significant difference at the blastocyst stage was found to be due to telomere DNA normalization between the cleavage and blastocyst stage of embryogenesis and not due to developmental arrest of embryos with short telomeres. Heterogeneity in telomere length within oocytes may provide an opportunity to improve the treatment of infertility through telomere-based selection of oocytes and embryos with reproductive competence.

## Author Summary

Human eggs (oocytes) are exceptionally prone to the erroneous acquisition of too few (monosomy) or too many (trisomy) chromosomes during development (meiosis). In fact, this type of instability, termed aneuploidy, represents the most common genetic cause of miscarriage in pregnant women (i.e. trisomy 16) and developmental delay in newborns (i.e. Down syndrome from trisomy 21). Although aneuploidy has become a growing problem for women as they delay childbearing into the late thirties, the underlying molecular etiology remains unknown. Since telomere DNA is known to protect the ends of chromosomes from degradation during cell division and is associated with aneuploidy in cancer cells in adults, we tested whether telomere DNA plays a role in aneuploidy development in human oocytes and embryos (where aneuploidy is much more common). We demonstrate that telomere DNA deficiency is indeed associated with aneuploidy in oocytes and early preimplantation (cleavage) stage embryos. This association is reversed upon development to late preimplantation (blastocyst) stage embryos as a result of telomere DNA elongation. These results indicate that telomere DNA deficiency may cause inappropriate chromosome segregation during human oocyte cell division (meiosis) and may serve as a marker for oocytes and embryos that lack the ability to produce healthy children.

## Introduction

Gain or loss of an entire chromosome (aneuploidy) is the most common genetic cause of miscarriage and developmental delay in humans. Advanced maternal age is a well known risk factor and a reflection of the observation that aneuploidy primarily arises during meiosis of the maternal gamete, the oocyte [1]. It is also well established that a decline in fertility occurs as maternal age increases. Therefore, as women continue to delay their childbearing into the mid to late thirties, there has been a growth in the utilization of preimplantation genetic screening (PGS) to avoid aneuploid conceptions during the in vitro fertilization (IVF)-based treatment of infertility. PGS of aneuploidy has recently advanced to include the ability to screen for all 24 chromosomes [2]–[4] has revealed that aneuploidy of any and all chromosomes found in humans can be present at the preimplantation stages of human embryonic development [5].

A number of events have been proposed to play a role in the development of aneuploidy during maternal meiosis of the oocyte. These include inappropriate or lack of formation of chiasmata, which link homologous chromosomes to ensure proper alignment [6], and late exit from the production line of oogenesis [7]. More recently, telomere dysfunction has been proposed as a phenomenon that unifies these and other events and as a general explanation for female reproductive senescence [8]. Indeed, the role of telomeres in maintaining chromosome stability was proposed over 70 years ago [9] and many studies have since demonstrated that excessive telomere shortening results in chromosome instability in somatic cells [10]. An animal model of telomere deficiency has also illustrated the importance of telomeres in germ cell chromosome stability [11]–[13]. In 4^th^ generation telomerase knockout mice, oocytes develop abnormal spindles. Since spindle formation is a critical event in proper chromosome segregation, this observation suggests that telomeres may play a role in the development of oocyte aneuploidy in the human. However, the prevalence of aneuploidy as a result of spindle formation defects in the telomerase knockout oocytes or ensuing embryos has not been specifically measured. In addition, artificially inducing telomere shortening through genetic deletion of the telomerase gene in mice may not reflect naturally occurring events in human oogenesis or embryogenesis.

In order to directly investigate whether telomere DNA is associated with development of aneuploidy in the preimplantation stages of human development, one must address several limitations. These include studying low quantities of DNA, obtaining access to normal human ooctyes and embryos, and developing the ability to simultaneously quantify telomere DNA and comprehensively diagnose aneuploidy from the same oocyte or embryo biopsy. The present study represents the first opportunity to overcome these hurdles. Much of this opportunity is related to the recent development of an accurate single cell SNP microarray based 24-chromosome aneuploidy screening methodology [2]. Since this technique involves the use of whole genome amplification (WGA), excess DNA is available to simultaneously quantify telomere DNA. Moreover, since SNP microarray based 24-chromosome aneuploidy screening has been employed in a number of Institutional Review Board approved clinical trials [3], [4], DNA from the highest quality (normal) IVF derived human oocytes and embryos is also available for analysis of telomere DNA. Therefore, the objectives of the present study were to first validate a method to simultaneously assess 24 chromosome aneuploidy and telomere DNA content from human oocytes and embryos and then to test the hypothesis that telomere DNA content is associated with development of human embryonic aneuploidy.

## Results

### Validation of relative telomere DNA quantitation after whole-genome amplification (WGA)

**Figure 1:**
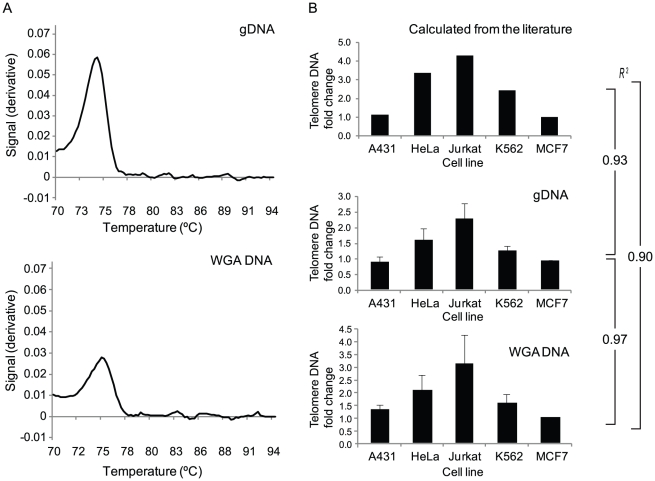
Valdiation of a novel assay for telomere DNA relative quantitation from limited amounts of starting material. (A) The telomere DNA assay amplification product produced a single peak upon dissociation curve analysis demonstrating specificity on purified genomic (g) DNA from a large quantity of cells, and from whole genome amplified (WGA) DNA from picogram quantities of purified gDNA. (B) Quantities of telomere DNA in various cancer cell lines relative to MCF-7 cells either reported in the literature or determined with the assay developed in this study. In each case, the Pearson correlation coefficient (R^2^) of the relative quantitation profiles are shown and illustrate the maintenance of relative abundance after characterization of picogram quantities of starting material.

A novel assay was developed and evaluated for the ability to accurately quantify relative amounts of telomere DNA from single or few cells after WGA. This was important to demonstrate the reliability of using existing 24-chromosome aneuploidy screened WGA DNA from oocyte and embryo biopsies. A multicopy Alu-Ya5 sequence was targeted as an endogenous control for telomere DNA quantity in order to accommodate differences in the number of input cells, the presence of aneuploidy, or possible single cell single locus PCR drop out within the reference and test samples. As expected, real-time PCR products using previously published primers for telomere DNA [14], and SYBR Green-based detection, displayed a single peak upon dissociation curve analysis (Figure 1A). This confirmed sequence specificity of amplification from both isolated genomic DNA and whole genome amplified DNA. In addition, the relative telomere DNA quantity observed in isolated genomic DNA and whole genome amplified DNA of various cell lines was similar to values calculated from the reported literature [15]–[19] (Figure 1B). Most importantly, the Pearson correlation (*r* ^2^) between experimentally determined relative telomere DNA quantities from isolated genomic DNA and whole genome amplified DNA from the same cell lines was 0.97, indicating that WGA faithfully represents the relative quantities of telomere DNA found in the original sample.

### Relative telomere DNA length in aneuploid and euploid polar bodies

**Figure 2:**
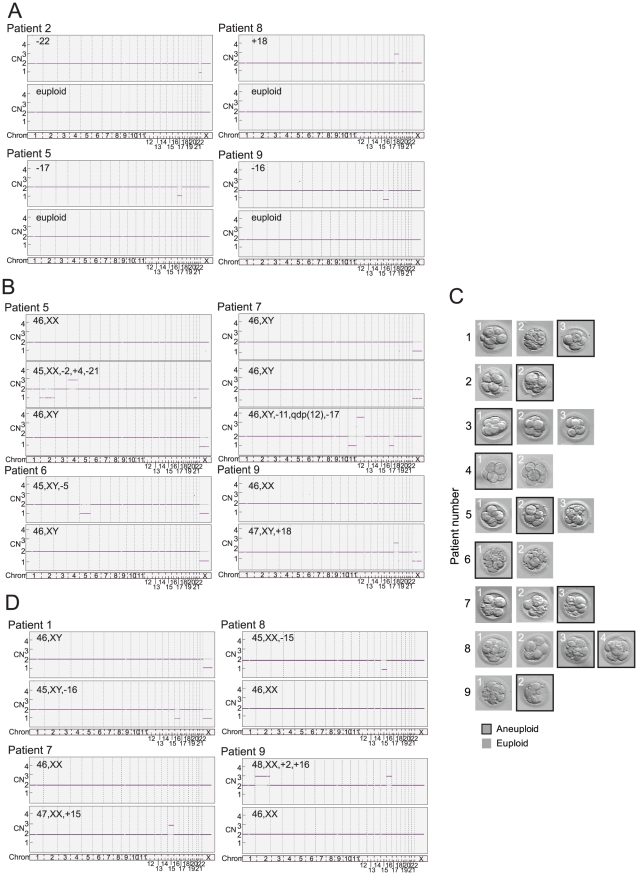
Example results of SNP microarray based 24 chromosome aneuploidy screening in human oocytes and preimplantation embryos that were also characterized for telomere DNA content. (A) SNP microarray based chromosome specific copy number graphs of euploid and aneuploid sibling polar bodies from polar body specific patients 2, 5, 8, and 9 (Table 1). (B) SNP microarray based copy number graphs of euploid and aneuploid sibling blastomeres from blastomere specific patients 5, 6, 7, and 9 (Table 2). (C) Photographs of cleavage stage sibling embryos from the 9 blastomere specific patients analyzed in the present study (Table 2). (D) SNP microarray based copy number graphs of euploid and aneuploid sibling blastocyst stage embryos from trophectoderm specific patients 1, 7, 8, and 9 (Table 3).

**Figure 3:**
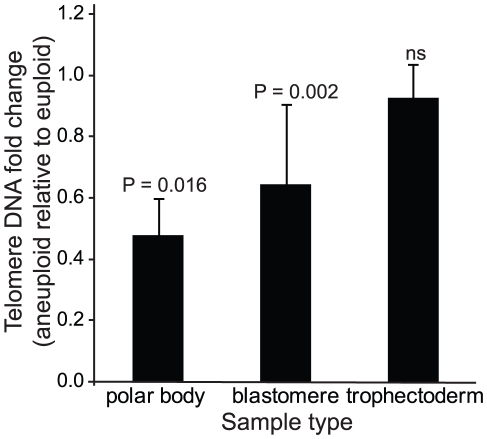
Mean quantities (±SEM) of telomere DNA found in aneuploid relative to euploid polar bodies, blastomeres, and trophectoderm biopsies. *P*-values from paired analysis of sibling samples within each patient are shown and illustrate the significant decrease in relative quantity in aneuploid polar bodies and blastomeres but not trophectoderm.

A total of 18 polar bodies (9 euploid and 9 aneuploid) from 9 IVF patients were evaluated for telomere DNA length (Table 1). The patient-specific variable of maternal age of the oocyte was controlled for by conducting paired analyses of telomere DNA quantity in sibling aneuploid and euploid polar body biopsies from oocytes derived from the same patient and IVF treatment cycle. Examples of results of aneuploidy screening are shown in Figure 2A. Aneuploid polar bodies displayed significantly lower quantities of telomere DNA than paired sibling euploid polar bodies (−3.04 fold, *P* = 0.016, Figure 3).

**Table 1:** Polar body samples evaluated by microarray based aneuploidy screening and relative telomere DNA quantitation.

Patient #	Maternal Age	sample type	Oocyte #	Aneuploidy	Oocyte #	Aneuploidy	telDNA fold change
**1**	32.2	PB2	1	−17	2	normal	0.44
**2**	41.3	PB2	1	−14,+16,+18	2	normal	0.04
**3**	34.5	PB2	1	−15	2	normal	1.02
**4**	36.2	PB2	1	+18	2	normal	0.09
**5**	33.3	PB2	1	−16	2	normal	0.69
**6**	41.3	PB1	1	−18,+21	2	normal	0.42
**7**	40.6	PB1	1	−22	2	normal	1.01
**8**	39.5	PB1	1	−15,−16	2	normal	0.32
**9**	33.3	PB1	1	+3,+7,+17	2	normal	0.32

Mean±SEM 0.43±0.12.

### Relative telomere DNA length in aneuploid and euploid blastomeres

A total of 24 blastomeres (14 euploid and 10 aneuploid) from 9 IVF patients were evaluated. Each patient provided at least one euploid and one aneuploid blastomere for paired analysis of relative telomere DNA length in sibling embryos (Table 2). Examples of results of aneuploidy screening are shown in Figure 2B. Again, all patient specific variables were controlled by comparing an aneuploid blastomere to a euploid blastomere from sibling embryos derived from the same patient and IVF treatment cycle. Samples were also evaluated by paired analysis for differences in embryo fragmentation, cell number, and morphological grade. Morphological characteristics were not significantly different between sibling aneuploid and euploid embryos in this study (Table 2). Photographs of each of the cleavage stage embryos included in this study are also shown in Figure 2C. Aneuploid blastomeres displayed significantly reduced telomere DNA quantity relative to their paired sibling euploid blastomeres (−2.6 fold, *P* = 0.002, Figure 3).

**Table 2:** Blastomere samples evaluated by microarray based aneuploidy screening and relative telomere DNA quantitation (aneuploid versus euploid).

Patient #	Maternal Age	Embryo no.	Cell no.	Fragmentation	Grade	Aneuploidy	Embryo no.	Cell no.	Fragmentation	Grade	Aneuploidy	telDNA Fold Change
**1**	33.6	1	8	5%	C	normal	3	6	5%	C	47,XY,+3	0.46
		2	8	30%	C	normal						
**2**	38.8	1	8	5%	B	normal	2	6	5%	C	45,XY,−21	0.22
**3**	33	2	8	5%	B	normal	1	10	5%	B	chaotic	0.21
		3	7	5%	B	normal						
**4**	33.8	2	8	5%	C	normal	1	8	5%	B	45,XY,−19	0.18
**5**	36.9	3	8	5%	C	normal	2	7	5%	C	45,XX−2,+4,−21	0.24
		1	8	5%	B	normal						
**6**	31.7	2	8	10%	C	normal	1	8	10%	B	45,XY,−5	1.89
**7**	38.5	1	6	15%	C	normal	3	6	30%	C	46,XY,−11,qdp(12),−17	0.19
		2	6	30%	C	normal						
**8**	39.5	1	8	5%	B	normal	3	8	5%	B	47,XY,+16	0.25
		2	8	10%	B	normal	4	8	10%	B	45,XY,−1,+21,−22	
**9**	32.3	1	8	10%	C	normal	2	7	10%	C	47,XY,+18	2.16
**Mean (±SEM)**			**7.6**	**10.4%**				**7.4**	**9.0%**			**0.65±0.26**

### Relative telomere DNA length in aneuploid and euploid trophectoderm

A total of 20 trophectoderm biopsies from sibling blastocyst stage embryos (10 aneuploid and 10 euploid) from 10 IVF patients were evaluated (Table 3). Examples of results of aneuploidy screening for each of the trophectoderm samples are shown in Figure 2D. In addition to controlling for maternal and paternal age by sibling paired analysis of telomere DNA quantity, embryo morphology was controlled for by selecting samples with identical morphological grade [20] within each pair of blastocysts (Table 3). Aneuploid blastocyst trophectoderm displayed quantities of telomere DNA that were not significantly different from their sibling euploid blastocysts (*P* = 0.340, Figure 3).

**Table 3:** Trophectoderm samples evaluated by microarray based aneuploidy screening and relative telomere DNA quantitation (aneuploid versus euploid).

Patient #	Maternal Age	Embryo no.	Grade	Aneuploidy	Embryo no.	Grade	Aneuploidy	telDNA Fold Change
**1**	32.3	1	4AA	normal	2	4AA	45,XY,−16	1.04
**2**	38.4	1	5BB	normal	2	5BB	47,XY,+16	0.84
**3**	39.5	2	4BB	normal	1	4BB	45,XY,−11,−20,+21	0.97
**4**	34.3	1	4BB	normal	2	4BB	47,XX,+15	0.72
**5**	31.9	2	5BB	normal	1	5BB	47,XX,+15	0.99
**6**	42.3	2	5AA	normal	1	5AA	47,XY,+9,+17,−20	0.22
**7**	31.9	1	5BB	normal	2	5BB	47,XX,+15	1.25
**8**	33.4	2	5BB	normal	1	5BB	45,XX,−15	1.51
**9**	40.0	2	4BB	normal	1	4BB	48,XX,+2,+16	0.92
**10**	35.7	1	5BB	normal	2	5BB	45,XY,−15	0.82

**Mean±SEM 0.93±0.11.**

### Telomere DNA elongation during human embryogenesis

**Figure 4:**
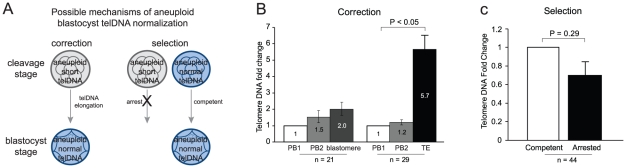
Telomere DNA in aneuploid blastocysts. (A) Diagram illustrating two potential mechanisms by which aneuploid blastocysts could obtain normalized quantities of telomere DNA. (B) Results of testing the potential for “correction” to explain normalized quantities of telomere DNA in aneuploid blastocysts. A significant increase (*P*<0.05) was found in telomere DNA quantity in trophectoderm from blastocysts relative to the 1^st^ polar body from the same oocyte. A significant increase was not found in blastomeres from cleavage stage embryos. This suggests that telomere DNA is reset between the cleavage and blastocyst stage of embryogenesis in humans. (C) Results of testing the potential for “selection” to explain normalized quantities of telomere DNA in aneuploid blastocysts. Significance was not reached when comparing sibling arrested and developmentally competent embryos (*P* = 0.29). This suggests that embryos with short telomeres aren't more prone to developmental arrest between the cleavage and blastocyst stage of embryogenesis in humans.

One mechanism by which aneuploid blastocysts may develop telomere DNA length equivalent to the length observed in euploid blastocysts is through telomere DNA elongation between the cleavage and blastocyst stages of development (Figure 4A). This phenomenon has been observed in animals [21], [22] and recently by unpaired analysis in humans [23]. Telomere DNA was quantified in the 1^st^ and 2^nd^ polar body and a blastomere from each of 21 cleavage stage embryos, and in the 1^st^ and 2^nd^ polar body and a trophectoderm biopsy from each of 29 blastocyst stage embryos. These samples were selected since all polar body and embryo biopsies were found to be euploid. Telomere DNA in each 2^nd^ polar body and each embryo biopsy was evaluated relative to the 1^st^ polar body that was derived from the same oocyte (Figure 4B). No significant differences in levels of telomere DNA were observed in 1^st^ or 2^nd^ polar bodies derived from the same oocyte (1.5-fold, *P* = 0.71, and 1.2-fold, *P* = 0.32, respectively). In addition, telomere DNA in blastomeres from cleavage stage embryos was not significantly different from levels found in the corresponding 1^st^ polar body (2.0-fold, *P* = 0.14). However, telomere DNA in trophectoderm biopsies from blastocyst stage embryos displayed a significant increase in quantity relative to the corresponding 1^st^ polar body (5.7-fold, *P* = 5.5×10^−10^). These results indicate that telomere DNA is elongated during development between the cleavage and blastocyst stage of development in the human.

### Relative telomere DNA length in developmentally arrested and competent embryos

Another mechanism by which aneuploid blastocysts may develop telomere DNA length equivalent to the length observed in euploid blastocysts is through developmental selection (Figure 4A). For this mechanism to be true, one would expect that embryos which fail to progress to the blastocyst stage (developmental arrest) would also possess significantly reduced telomere DNA length relative to embryos which successfully develop to the blastocyst stage (developmentally competent). Forty-four paired analyses of developmentally arrested and competent embryos, that were otherwise developmental stage and ploidy matched, were evaluated for relative telomere DNA quantity (Figure 4C). A significant difference in telomere DNA length between arrested and developmentally competent embryos was not observed (*P* = 0.29). This result indicates that aneuploid blastocysts do not develop levels equivalent to euploid blastocysts through selection against embryos with short telomeres.

## Discussion

An accurate method for simultaneous relative quantitation of telomere DNA content and 24 chromosome aneuploidy screening in single cells was developed. Key features of this assay include the use of a multicopy endogenous control target sequence to prevent differences in the number of cells, nuclear DNA content (haploid bivalent, haploid univalent, diploid), the presence of aneuploidy, or single locus drop out from impacting the relative quantitation of telomere DNA in the test and reference samples. The use of a multicopy gene for normalization may also help to avoid issues with amplification bias from single cells since it represents an abundant target within the single cell and would be less susceptible to the well characterized single locus bias that is found after applying WGA [24]. The same concept may be true when applying WGA based amplification to evaluate telomere DNA since it also represents a multicopy sequence. It was also important to utilize DNA processing specific relative quantitation since an apparent decrease in telomere DNA content relative to the total amount of DNA was observed after whole genome amplification (mean ΔC_T_ of 22.5±1.3) compared to isolated genomic DNA (mean ΔC_T_ of 12.9±0.6) from the same cell lines. In addition, the paired nature of the relative quantitation was also important. For example, the paired sample study design controlled for all patient specific variables that may be associated with telomere DNA content including maternal age, paternal age, and genetic background. Paired analysis may also be important to avoid potential issues related to the use of the Alu-Ya5 sequence when normalizing telomere DNA content measurements since different individuals in a given population may possess natural variations in Alu-Ya5 copy number. The paired-sample design also controls for all unknown patient specific variables inherent in any study involving humans.

In addition to controlling for differences that may be attributed to patient specific variables, this study also controlled for morphological characteristics of cleavage and blastocyst stage embryos. This was important since previous work indicated that embryo fragmentation was predictive of telomere DNA quantity [25]. The present study established that pairs of euploid and aneuploid samples were either not significantly different (cleavage stage) or specifically selected to have been given identical morphological grades (blastocyst stage). With these parameters controlled within each paired analysis, significant difference in telomere DNA quantity was observed between the aneuploid and euploid polar bodies and cleavage stage blastomeres but not trophectoderm from the blastocyst. The correction of telomere DNA length through elongation between the cleavage and blastocyst stage of embryogenesis was also established in this study. Similar findings were obtained in animal studies [21], [22] and in a recent study involving unpaired analyses of human embryos [23].

Most importantly, results of the present study demonstrate that telomere DNA length is associated with human aneuploidy development for the first time. The correlation between telomere length and aneuploidy during embryogenesis also corresponds with the previously identified and predominantly maternal meiotic origin of aneuploidy [1], and with recent observations of genomic instability at the cleavage stage of development [26]. Interestingly, the relationship between telomere content and aneuploidy was maintained when embryonic cells were evaluated at the cleavage stage of development despite the presence of both maternal and paternal chromosome telomere DNA. However, the extent to which telomere content was decreased in aneuploid cleavage stage embryonic cells was slightly lower than that observed in polar bodies from the oocyte (Figure 3), which is consistent with the maternal origin of aneuploidy. Future studies may need to focus on whether there is an association between telomere DNA content and the cell division and parental specific origins of aneuploidy. This will require development of the ability to predict origins of aneuploidy in single cell quantities of DNA in a manner similar to that described for analysis of large quantities of DNA from products of conception [27]. Although rare, it is possible for compensation of meiosis I errors derived from premature separation of sister chromatids (PSSC) to lead to a euploid oocyte by segregation of the abnormality to the 2^nd^ polar body [28]. However, all the aneuploid polar bodies evaluated in this study were only from oocytes which led to aneuploid embryos. Future studies might include evaluating whether decreased telomere length is differentially associated with aneuploidy from PSSC compared to nondisjunction.

Since reduced recombination remains one of the well known risk factors in nondisjunction and development of aneuploidy in humans [6], the strong association between telomere length and aneuploidy in oocytes and cleavage stage embryos in the present study might be the result of reduced meiotic recombination and impaired chromosome pairing and synapsis that have been observed in oocytes from telomere deficient mice [12]. In support of this is the observation that the decrease in oocyte telomere length reported in telomere deficient mice relative to wild type oocytes was approximately 0.4-fold (20/50, as displayed in figure 5b of reference [12]), similar to the decrease of 0.43-fold observed in aneuploid relative to euploid polar bodies in the present study (Figure 3). Some studies also suggest that telomeres are directly involved in homologous chromosome pairing where synapsis may begin at the telomeres [29]. Further investigation into whether polar bodies and embryos with decreased telomere length also possess reduced numbers of recombination events may help support these findings.

The observation that heterogeneity in telomere DNA content exists within oocytes and embryos derived from a single controlled ovarian stimulation (COS) cycle may represent a phenomenon relevant to improving reproductive medicine. If reduced telomere DNA length represents an intermediate event that precedes aneuploidy development, then telomere length may represent a useful marker of embryonic reproductive potential. Given the recent evidence that not all euploid embryos possess reproductive potential, that the age related decline in fertility is not entirely due to aneuploidy [3], and the results of the present study, further investigation into the predictive value of telomere DNA length for ovarian reserve and reproductive potential and senescence using the methods developed in this study is warranted.

## Materials and Methods

### Ethics statement

This study was conducted under Institutional Review Board approval from Western IRB (Olympia, WA) and with informed patient consent.

### Relative telomere measurement by quantitative real-time PCR

Telomere DNA was amplified using “telg” and “telc” primers previously described by Cawthon *et al* [14] at a final concentration of 900 nM each. SYBR Green PCR Master Mix (Applied Biosystems Inc., Foster City, CA) was used at the manufacturer's recommended concentration. In order to normalize input DNA, primers and a TaqMan probe for the multicopy Alu (Ya5 family) sequence were used as previously described [30] but with use of a FAM dye instead of VIC (Applied Biosystems Inc.). A multicopy gene for normalization was used to avoid potential issues with a single copy gene locus dropout from single cell WGA, and the potential impact of aneuploidy on single copy gene copy number. TaqMan Alu primers and probe, and TaqMan Gene Expression Master Mix were used at the manufacturer's recommended concentrations (Applied Biosystems Inc.). Both the telomere DNA SYBR Green and Alu DNA TaqMan assay reactions were performed in quadruplicate for each template DNA and in a final reaction volume of 5 µl in a MicroAmp Optical 384-Well Reaction Plate (Applied Biosystems Inc.). Five ng of genomic DNA or 10 ng of WGA DNA template was used in each reaction. A 7900HT SDS real-time PCR instrument (Applied Biosystems Inc.) was used with the default cycling conditions and dissociation curve settings in the instrument control and data acquisition software (SDS version 2.3, Applied Biosystems Inc.). The default settings of RQ Manager version 1.2 data analysis software (Applied Biosystems Inc.) were used to assign a threshold cycle number to each reaction. Results were exported to Excel (Microsoft Inc., Redmond, WA) for statistical analysis.

### Cell line DNA standards for assay validation

MCF-7, HeLa, A431, Jurkat, and K562 cell line isolated genomic DNA was obtained from BioChain Inc. (Hayward, CA) and the expected telomere DNA length was estimated from previous reports [15]–[19]. More specifically, the average length in kilobases (kb) reported for each cell line was used as the numerator (i.e. A431 = 3.0 kb; K562 = 6.5 kb; HeLa = 9.0 kb; and Jurkat = 11.5 kb), and the average length in the MCF-7 cell line was used as the denominator to calculate a ratio (relative fold change quantity). The resulting relative fold change quantities served as a reference for analysis using the assay developed in the present study. Whole genome amplification (WGA) was performed on 35 picograms of genomic DNA using a GenomePlex Single Cell WGA4 Kit according to the manufacturer's instructions (Sigma Aldrich Inc., St. Louis, MI). WGA DNA was purified using GenElute PCR purification kit according to the manufacturer's instructions (Sigma Aldrich Inc.). Purified WGA DNA was quantified using a Nanodrop 8000 spectrophotometer (Nanodrop Inc., Wilmington, DE).

Relative quantitation (fold change) of telomere DNA content was determined using the comparative C_T_ method [31] with telomere DNA representing the “gene of interest” and Alu DNA representing the “internal control” (i.e. telomere DNA C_T_−Alu DNA C_T_ = ΔC_T_). Cell line telomere DNA ΔC_T_ values were evaluated with the MCF-7 cell line ΔC_T_ as the reference control. Pearson correlation coefficients were calculated for fold change values experimentally determined from either genomic DNA (n = 4) or WGA DNA (n = 4) with respect to the expected fold change values calculated from telomere DNA lengths reported in the literature [15]–[19]. A Pearson correlation coefficient was also calculated for experimentally determined genomic DNA with respect to WGA DNA. It should be noted that the assay developed in this study cannot be used to determine an absolute measure of telomere length such as might be represented in kb units. The assay is only capable of determining the relative quantity of telomere DNA in one sample compared to another (fold change).

### Aneuploid and euploid polar body and embryo biopsy DNA for telomere quantitation

DNA from human polar bodies and embryo biopsy WGA was obtained as previously described [32], [33], and used for analysis of telomere DNA content. All samples were obtained under Institutional Review Board approval (WIRB, Olympia, WA) and with informed patient consent. Samples were specifically selected from cases where both an aneuploid and a euploid 1^st^ polar body, 2^nd^ polar body, or embryo biopsy WGA DNA sample was available from the same patient and IVF treatment cycle, and from embryos with similar morphology (cell number, fragmentation, and grade), in order to allow for paired analysis. All polar bodies were biopsied from oocytes that led to embryos suitable for transfer by conventional morphological assessment. Euploid polar bodies used in this study were always associated with oocytes in which both the 1^st^ and 2^nd^ polar body was euploid. Likewise, aneuploid polar bodies used in this study were always associated with oocytes which produced an aneuploid embryo.

Euploid embryo or polar body ΔC_T_ values were used as the reference control for aneuploid embryo or polar body ΔC_T_ values in patient specific pairs. Confirmation of normal distribution of aneuploid or euploid embryo or polar body telomere DNA quantities was evaluated by performing a Shapiro-Wilk W test. Telomere DNA length (ΔC_T_) in polar bodies, blastomeres, trophectoderm biopsies, and arrested embryos was compared for significance using a paired Student's t-test. Blastocyst stage embryo morphology was controlled by selecting samples with identical morphological grade [20] within each patient specific pair. Aneuploid and euploid cleavage stage embryo telomere DNA length (ΔC_T_) was compared using a mixed linear model with fixed effects terms from telomere DNA length (ΔC_T_) versus ploidy state, and a patient random effect term to account for between-patient variability. Cleavage stage embryo telomere DNA lengths were also tested in a mixed linear model against characteristics of morphology, including cell number, fragmentation rate, and embryo grade. Tests were performed with R's nlme package [34], [35]. Telomere results were analyzed blind to aneuploidy status and vice-versa.

Aneuploidy assignments were based on the use of a previously published method involving single nucleotide polymorphism (SNP) microarray based copy number analysis [2]. This method has had preclinical validation performed on randomized and blinded single cells from cell lines with previously well characterized aneuploid karyotypes, demonstrating 98.6% accuracy and no false positive aneuploidy diagnoses [2]. It has also had clinical validation performed through a prospective randomized non-selection clinical trial demonstrating 100% negative predictive value for the reproductive potential of human embryos [3]. SNP microarray data described in this study have been deposited in NCBI's Gene Expression Omnibus and are accessible through GEO Series accession number GSE25864 (http://www.ncbi.nlm.nih.gov/geo).

### Evaluation of putative mechanisms of telomere DNA normalization in blastocysts with aneuploidy

Twenty one sets of samples including the 1^st^ polar body, 2^nd^ polar body, and blastomere derived from the same oocyte, and 29 sets of samples inluding the 1^st^ polar body, 2^nd^ polar body, and trophectoderm, were evaluated. All samples were included on the basis of chromosomal normalcy as predicted using SNP microarray based analysis described above. Quantities of telomere DNA in the 2^nd^ polar bodies, blastomeres, and trophectoderm were calculated relative to the corresponding first polar body from the same original oocyte. Forty four embryos that arrested at developmental stages equivalent to 44 developmentally competent sibling embryos were evaluated. Quantities of telomere DNA in arrested embryos were calculated relative to the developmentally competent embryos.
